# Combining targeted metabolite analyses and transcriptomics to reveal the specific chemical composition and associated genes in the incompatible soybean variety PI437654 infected with soybean cyst nematode HG1.2.3.5.7

**DOI:** 10.1186/s12870-021-02998-4

**Published:** 2021-05-14

**Authors:** Xue Shi, Qiansi Chen, Shiming Liu, Jiajun Wang, Deliang Peng, Lingan Kong

**Affiliations:** 1 State Key Laboratory for Biology of Plant Diseases and Insect Pests, Institute of Plant Protection, Chinese Academy of Agricultural Sciences, Beijing, 100193 China; 2 Zhengzhou Tobacco Research Institute of CNTC, Zhengzhou, Henan China; 3 Soybean Research Institute, Heilongjiang Academy of Agricultural Sciences, Harbin, 150086 China

**Keywords:** Incompatible and compatible soybean varieties, Soybean cyst nematode, Metabolomic analyses, Transcriptomics, Combination analyses

## Abstract

**Background:**

Soybean cyst nematode, *Heterodera glycines*, is one of the most devastating pathogens of soybean and causes severe annual yield losses worldwide. Different soybean varieties exhibit different responses to *H. glycines* infection at various levels, such as the genomic, transcriptional, proteomic and metabolomic levels. However, there have not yet been any reports of the differential responses of incompatible and compatible soybean varieties infected with *H. glycines* based on combined metabolomic and transcriptomic analyses.

**Results:**

In this study, the incompatible soybean variety PI437654 and three compatible soybean varieties, Williams 82, Zhonghuang 13 and Hefeng 47, were used to clarify the differences in metabolites and transcriptomics before and after the infection with HG1.2.3.5.7. A local metabolite-calibrated database was used to identify potentially differential metabolites, and the differences in metabolites and metabolic pathways were compared between the incompatible and compatible soybean varieties after inoculation with HG1.2.3.5.7. In total, 37 differential metabolites and 20 KEGG metabolic pathways were identified, which were divided into three categories: metabolites/pathways overlapped in the incompatible and compatible soybeans, and metabolites/pathways specific to either the incompatible or compatible soybean varieties. Twelve differential metabolites were found to be involved in predicted KEGG metabolite pathways. Moreover, 14 specific differential metabolites (such as significantly up-regulated nicotine and down-regulated D-aspartic acid) and their associated KEGG pathways (such as the tropane, piperidine and pyridine alkaloid biosynthesis, alanine, aspartate and glutamate metabolism, sphingolipid metabolism and arginine biosynthesis) were significantly altered and abundantly enriched in the incompatible soybean variety PI437654, and likely played pivotal roles in defending against HG1.2.3.5.7 infection. Three key metabolites (N-acetyltranexamic acid, nicotine and D,L-tryptophan) found to be significantly up-regulated in the incompatible soybean variety PI437654 infected by HG1.2.3.5.7 were classified into two types and used for combined analyses with the transcriptomic expression profiling. Associated genes were predicted, along with the likely corresponding biological processes, cellular components, molecular functions and pathways.

**Conclusions:**

Our results not only identified potential novel metabolites and associated genes involved in the incompatible response of PI437654 to soybean cyst nematode HG1.2.3.5.7, but also provided new insights into the interactions between soybeans and soybean cyst nematodes.

**Supplementary Information:**

The online version contains supplementary material available at 10.1186/s12870-021-02998-4.

## Background

Soybean cyst nematode (SCN), *Heterodera glycines*, is one of the most devastating pathogens of soybean and causes a large annual yield losses worldwide [1–4]. SCN is a typical obligate endoparasitic nematode [3]. The juveniles of SCN pierce the soybean root with a spear-like feeding structure called a stylet to penetrate the soybean root [3, 5]. They then invade soybean roots and migrate to the vascular bundles, where they establish a complex feeding site known as a syncytium [6, 7]. The formation and maintenance of the syncytium lead to dramatic changes in the internal root structure, affect the root system and cause damage [3]. Additionally, soybean roots produce many defense-related compounds, such as reactive oxygen species, and activate hormone signaling pathways in response to nematode infection, and great changes in metabolites are observed in soybean roots upon SCN infection [8, 9].

Recently, the use of “omics” technology to obtain biological data has surged, and the most commonly used omics approaches are genomics, transcriptomics, proteomics and metabolomics [10–12]. There are numerous research reports addressing soybean-SCN interactions on the basis of genomic and transcriptomic analyses [4, 13–20]. The genomes of SCN and many soybean varieties have been evaluated to further investigate their biology, characteristics and potential interactions [4, 19, 21–24]. Many candidate soybean resistance loci and/or genes have been identified based on the genome-wide association studies (GWAS) [13, 18, 20]. Moreover, many studies on the expression profiling of different plant tissues, such as the whole root samples and the syncytium structure, have been carried out in compatible and incompatible soybean varieties infected with SCN, and many differentially expressed genes (DEGs) and related KEGG pathways have been predicted [14, 15, 17, 25, 26].

Metabolomics is used to characterize metabolic patterns and further study the functions and significance of key differential metabolites that play important roles in biological and medical fields [27–29]. Plant metabolomics has gained increasing attention and has become a mature method for studying plant responses to both biotic and abiotic stresses. Therefore, plant metabolomics is considered as an indispensable tool in the study of plant-pathogen interactions [30]. Plants respond to pathogen infection by producing defense-related metabolites, especially the plant secondary metabolites, which play important roles in plant resistance [31]. Metabolomics can provide technical support for the identification of plant metabolites, which are potential sources of new compounds for nematode control [32]. Research on plant nematodes has become increasingly abundant, especially work addressing root-knot nematodes. Eloh et al. performed metabolomic analysis of root-knot nematodes treated with maleimide and proved that maleimide could be used as a new potential nematicide [33]. In addition, they studied the levels of metabolites in tomato plants after root-knot nematode infection, and found that tomato roots showed changes in biochemical pathways in response to root-knot nematode infection [33]. Recently, Kantor et al. investigated the metabolite profiles of both incompatible and compatible watermelon accessions with root-knot nematode infection and found that the roots of incompatible wild watermelon accessions were rich in metabolic compounds that had a nematicidal effect but these compounds showed decreased levels in commercial watermelon cultivars [31]. However, there have been a few studies on soybean-SCN interactions based on proteomic and metabolomic analyses [34, 35]. Differential proteins and metabolites between incompatible and compatible soybean roots were identified with the traditional approaches more than ten years ago [34]. Recently, Kang et al. studied the effect of *Bacillus simplex* strain Sneb545 on soybean secondary metabolites under SCN infection by combining advanced and high-throughput approaches of transcriptomic and metabolomic analyses and found that Sneb545-treated soybeans showed higher concentrations of various nematicidal metabolites [35].

To date, there have been no reports on the differences in metabolic pathways between incompatible and compatible soybean varieties infected with soybean cyst nematodes. In this study, an incompatible soybean variety PI437654 [36–38] and three compatible soybean varieties, Williams 82 (WM82), Zhonghuang 13 (ZH13) and Hefeng 47 (HF47), were selected as the research materials, and LC/MS full-scan detection technology was used to investigate the differences in metabolites and metabolic pathways between the incompatible and compatible soybean varieties after inoculation with HG1.2.3.5.7. Metabolites overlapped between the incompatible and compatible soybean varieties, differential metabolites specific to the incompatible or compatible soybean varieties and the potential KEGG metabolic pathways of these metabolites were identified. Moreover, the genes associated with the significantly up-regulated differential metabolites were predicted, and the associated genes were classified into two types. One type of the genes were associated with significantly up-regulated differential metabolites specific to the incompatible soybean variety PI437654, and the other type of the genes were associated with the overlapping differential metabolites that were significantly up-regulated in the incompatible soybean variety PI437654 but simultaneously significantly down-regulated in the three compatible soybean varieties. The results not only identified potential novel metabolites and associated genes involved in the incompatible response of PI437654 to soybean cyst nematode HG1.2.3.5.7, but also provided new insights into the interaction between soybeans and soybean cyst nematodes.

## Results

### Differentiation of the metabolites between incompatible and compatible soybean varieties under infection with HG1.2.3.5.7

**Fig. 1:**
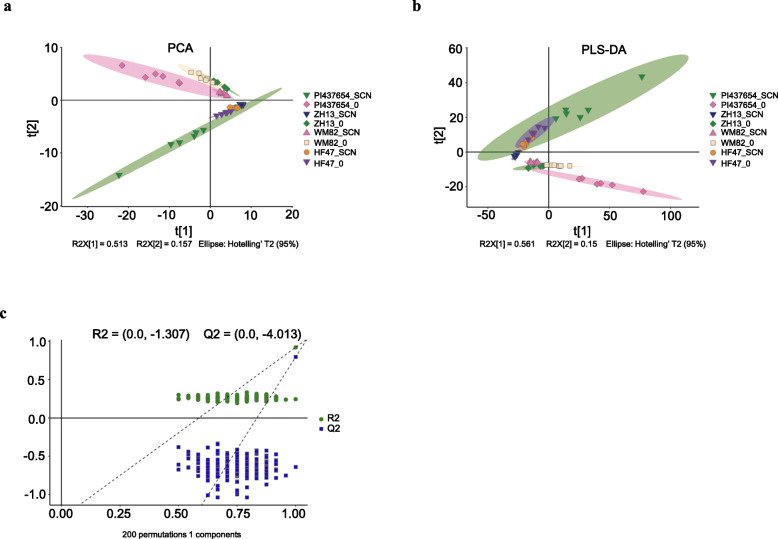
Principal component analysis (PCA), partial least-squares-discriminant analysis (PLS-DA) and permutation tests of metabolite profiling data. **a** PCA score plots of the incompatible soybean variety PI437654 and the three compatible soybean varieties, Williams 82 (WM82), Zhonghuang 13 (ZH13) and Hefeng 47 (HF47), infected with HG1.2.3.5.7. **b** PLS-DA score plots based on the metabolite profiling data of the incompatible soybean variety PI437654 and the three compatible soybean varieties, WM82, ZH13 and HF47, infected with HG1.2.3.5.7. **c** Permutation tests of PLS-DA models. The permutation tests were carried out with 200 random permutations. Each point represents the metabolite profile of one biological replicate

To investigate the differential root metabolites between the incompatible and compatible soybean varieties, the root samples of the incompatible soybean variety PI437654 and three compatible soybean varieties (WM82, ZH13 and HF47) (Fig. S1, Table S1) infected with SCN HG1.2.3.5.7 (‘_SCN’) at 8 dpi were collected, and their corresponding control root samples inoculated with water (‘_0’) were also collected, respectively. All the root samples were subjected to metabolomic analyses. In the PCA chart of incompatible soybean, all six replicate samples of PI437654_SCN were clustered together, and all six replicate samples of PI437654_0 were also clustered together (Fig. 1a and S2). Moreover, the PI437654_SCN samples were clearly separated from those of PI437654_0 (Fig. 1a and S2), which suggested that there were significant changes in metabolites in the incompatible soybean variety PI437654 after inoculation with HG1.2.3.5.7. The PLS-DA results were similar to those of the PCA, and the samples of PI437654_SCN were also clearly separated from those of PI437654_0 (Fig. 1b and S2). The PCA and PLS-DA results showed that all three compatible soybean varieties, WM82_SCN, ZH13_SCN and HF47_SCN, were dramatically separated from WM82_0, ZH13_0, and HF47_0, respectively (Fig. 1a, b and S2), which indicated that the inoculation of HG1.2.3.5.7 caused obvious changes in the root metabolites of the three compatible soybean varieties. Furthermore, according to the cumulative interpretation rate of the model for the incompatible and compatible soybean varieties (Fig. 1c, Table S2), both the R^2^ and Q^2^ values were close to 1, clearly indicating that the predictive power and quality of the two groups of models were suitable for the subsequent experiments. These results indicated that the inoculation of HG1.2.3.5.7 caused significant metabolic changes in both the incompatible soybean variety PI437654 and the three compatible soybean varieties.

### Identification of differential metabolites in incompatible and compatible soybean varieties

In total, 19 significantly differential metabolites were screened from the incompatible soybean variety PI437654 infected with HG1.2.3.5.7 at 8 dpi compared to that in the corresponding control of PI437654 inoculated with water (designated PI437654_SCN vs PI437654_0), among which seven metabolites were up-regulated, and 12 metabolites were down-regulated (Table 1). In the compatible soybean WM82_SCN vs WM82_0, 17 obviously differential metabolites were identified, including three up-regulated and 14 down-regulated metabolites (Table 1). Similarly, there were 12 dramatically differential metabolites in ZH13_SCN vs ZH13_0, all of which were down-regulated (Table 1). There were 17 dramatically differential metabolites in HF47_SCN vs HF47_0, including three up-regulated metabolites and 14 down-regulated metabolites (Table 1). The differential metabolites among the four soybean varieties exhibited different profiles, which were mainly divided into the following three categories.

1. Overlapping differential metabolites between the incompatible soybean variety PI437654 and the three compatible soybean varieties. There were four overlapping differential metabolites (Table 1, Fig. S3), including D-leucine, D,L-tryptophan, 16-hydroxyhexadecanoic acid and linolenic acid, which were amino acids and fatty acids. Linolenic acid was simultaneously dramatically down-regulated in the incompatible soybean variety PI437654_SCN vs PI437654_0 and the three compatible soybean varieties. D-leucine was dramatically down-regulated in the incompatible soybean variety PI437654_SCN vs PI437654_0 and two compatible soybean varieties of ZH13_SCN vs ZH13_0 and HF47_SCN vs HF47_0, but was slightly up-regulated in the compatible soybean variety of WM82_SCN vs WM82_0. 16-Hydroxyhexadecanoic acid was dramatically down-regulated in the incompatible soybean variety PI437654_SCN vs PI437654_0 and two compatible soybean varieties of WM82_SCN vs WM82_0 and ZH13_SCN vs ZH13_0, but was obviously up-regulated in the compatible soybean variety of HF47_SCN vs HF47_0 (Table 1, Fig. S3). In contrast, D,L-tryptophan was simultaneously down-regulated in the three compatible soybean varieties but significantly up-regulated in the incompatible soybean variety PI437654_SCN vs PI437654_0 (Table 1). The results indicated that D,L-tryptophan had a role in defense metabolite production in the incompatible soybean variety PI437654 infected by HG1.2.3.5.7.
2. Specific differential metabolites in the compatible soybean varieties. There were 18 significantly differential metabolites that were specifically present in the compatible soybean varieties and absent in the incompatible soybean variety PI437654_SCN vs PI437654_0 (Table 1, Fig. S3). Both 2-oxo-4-methylthiobutanoic acid and 4-hydroxycoumarin were simultaneously overlapped and down-regulated in all the three compatible soybean varieties (Table 1, Fig. S3), while the other 16 metabolites were present in only one or two of the three compatible soybean varieties (Table 1, Fig. S3). Prunetin was specific to WM82_SCN vs WM82_0 and dramatically up-regulated, while trans-ferulic acid was specific to HF47_SCN vs HF47_0 and dramatically up-regulated (Table 1, Fig. S3). 3-Hydroxy-7-methoxyflavone was dramatically up-regulated in WM82_SCN vs WM82_0 but dramatically down-regulated in ZH13_SCN vs ZH13_0, while PC(O-14:0/2:0) was dramatically up-regulated in HF47_SCN vs HF47_0 but dramatically down-regulated in WM82_SCN vs WM82_0 (Table 1, Fig. S3). The other 14 metabolites were obviously down-regulated. N-cyclohexanecarbonylpentadecylamine, isoliquiritigenin and drimenol were simultaneously down-regulated in WM82_SCN vs WM82_0 and ZH13_SCN vs ZH13_0, while dichotosinin was simultaneously down-regulated in WM82_SCN vs WM82_0 and HF47_SCN vs HF47_0 (Table 1, Fig. S3). Rhamnazin and betulinic acid were specific to WM82_SCN vs WM82_0, baicalein was specific to ZH13_SCN vs ZH13_0, while ent-kaur-16-en-19-ol, isopimaric acid, communic acid, hydroxy citronellal and hexadecanamide were specific to HF47_SCN vs HF47_0 (Table 1, Fig. S3). The down-regulated differential metabolites in the compatible soybean varieties were probably linked to basal defenses or physiological responses modulated by HG1.2.3.5.7 infection for the establishment and development of the feeding sites.
3. Specific differential metabolites in the incompatible soybean variety PI437654. There were 10 differential metabolites specific to the incompatible soybean variety PI437654_SCN vs PI437654_0 (Table 1, Fig. S3), including seven down-regulated and three up-regulated metabolites (Table 1). D-Aspartic acid and linoleic acid were the top two significantly down-regulated metabolites, showing an approximately 70-fold reduction in PI437654_SCN vs PI437654_0 (Table 1), while N-palmitoyl alanine, cycloleucine and D,L-2,4-diaminobutyric acid were reduced approximately 8-, 3- and 2.5-fold, respectively, in PI437654_SCN vs PI437654_0 (Table 1). Among the three up-regulated metabolites, N-acetyltranexamic acid and nicotine were more abundant by approximately 12.9- and 5.8-fold in PI437654_SCN vs PI437654_0 (Table 1). In addition, five obviously differential metabolites, including two down-regulated (pipecolinic acid and gallocatechin gallate) and three up-regulated (4-methylquinoline, nicotyrine and L-trans-4-hydroxy-L-proline) metabolites were identified in PI437654_SCN vs PI437654_0. However, these five metabolites were not uniquely present in the incompatible soybean variety PI437654 and were also present in the compatible soybean varieties (Table 1). These results indicated that up-regulated N-acetyltranexamic acid and nicotine might play potential roles in defense against HG1.2.3.5.7 infection in the incompatible soybean variety PI437654.

**Table 1:** Differential metabolites in the roots of the incompatible soybean variety PI437654 and three compatible soybeans, WM82, ZH13 and HF47, infected with the HG1.2.3.5.7

Metabolite	PI 437654	WM82	ZH13	HF47
linolenic acid	−1.604^a^	−1.254	− 1.741	−1.172
D-leucine	−0.759	0.322	−2.313	−1.289
16-hydroxyhexadecanoic acid	−1.100	−0.537	−1.138	0.685
D,L-tryptophan	1.300	−0.544	−0.892	− 0.556
D-aspartic acid	−8.796	/	/	/
linoleic acid	−6.199	/	/	/
N-palmitoyl alanine	−3.060	/	/	/
cycloleucine	−1.632	/	/	/
D,L-2,4-diaminobutyric acid	−1.307	/	/	/
phytosphingosine	−0.987	/	/	/
10-oxo-nonadecanoic acid	−0.885	/	/	/
N-acetyltranexamic acid	3.688	/	/	/
nicotine	2.543	/	/	/
L-arginine	0.893	/	/	/
pipecolinic acid	−2.026	−1.591	−1.390	/
gallocatechin gallate	−1.688	− 1.573	/	/
4-methylquinoline	1.110	/	/	−0.584
nicotyrine	1.096	/	/	−0.563
L-trans-4-hydroxy-L-proline	1.047	/	/	−0.556
2-oxo-4-methylthiobutanoic acid	/	−0.852	−1.311	−0.474
4-hydroxycoumarin	/	−0.933	−1.293	−0.430
N-cyclohexanecarbonylpentadecylamine	/	−2.816	−4.143	/
PC(O-14:0/2:0)	/	−2.613	/	1.522
isoliquiritigenin	/	−2.000	−3.391	/
rhamnazin	/	−1.037	/	/
betulinic acid	/	−1.020	/	/
drimenol	/	−0.923	−2.296	/
3-hydroxy-7-methoxyflavone	/	2.170	−7.687	/
prunetin	/	1.637	/	/
baicalein	/	/	−1.560	/
ent-kaur-16-en-19-ol	/	/	/	−5.012
isopimaric acid	/	/	/	−3.091
communic acid	/	/	/	−2.529
hydroxy citronellal	/	/	/	−1.470
hexadecanamide	/	/	/	−1.324
dichotosinin	/	−2.044	/	−1.212
trans-ferulic acid	/	/	/	3.011

*Note*: ‘^a^’ represents the log_2_ FC value, which is the ratio of the average relative expression of metabolites in the incompatible soybean variety PI437654 and the three compatible soybean varieties, WM82, ZH13 and HF47, infected by HG1.2.3.5.7 compared with that in the controls innoculated with water. A positive value indicates that the differential metabolites are up-regulated, while a negative value indicates the differential metabolites are down-regulated. ‘/’ indicates that such metabolites were absent in the tested soybean varieties

### Metabolic pathways of the differential metabolites in the incompatible and compatible soybean varieties

**Fig. 2:**
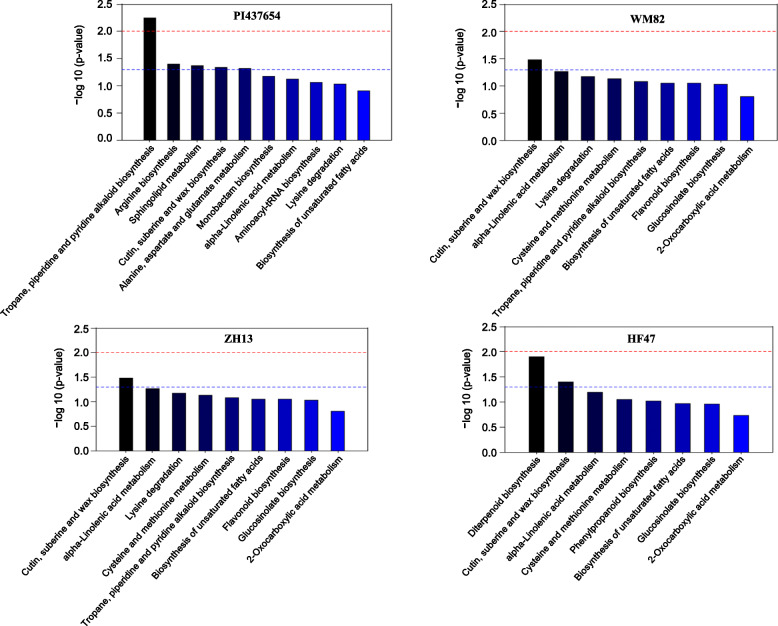
KEGG pathway analyses of differential metabolites in the incompatible soybean variety PI437654 and the three compatible soybean varieties, WM82, ZH13 and HF47. The threshold of a *p*-value< 0.01 is indicated by the red line, while the threshold of a *p*-value< 0.05 is indicated by the blue line. KEGG metabolic pathways were considered to be significantly altered when the corresponding *p*-value was above the blue line or the red line

The metabolic pathways of differential metabolites were analyzed by KEGG database searching. In total, 14 metabolic pathways were altered in the incompatible soybean variety PI437654_SCN vs PI437654_0, while nine, nine and eight metabolic pathways were altered in the compatible soybean varieties, WM82_SCN vs WM82_0, ZH13_SCN vs ZH13_0, HF47_SCN vs HF47_0, respectively (Fig. 2, Table 2). The incompatible response-related KEGG pathways were the focuses of this study and were divided into the following two categories.

1. Overlapping KEGG metabolic pathways between the incompatible and compatible soybean varieties. KEGG metabolic pathways including cutin, suberine and wax biosynthesis (ath00073), alpha-linolenic acid metabolism (ath00592) and the biosynthesis of unsaturated fatty acids (ath01040) were enriched and overlapped between the incompatible soybean variety PI437654 and the three compatible soybean varieties according to association with two differential metabolites, 16-hydroxyhexadecanoic acid and linolenic acid (Table 2). Moreover, the cutin, suberine and wax biosynthesis KEGG metabolic pathway (ath00073) was obviously activated in both the incompatible soybean variety PI437654 and the three compatible soybean varieties infected with HG1.2.3.5.7 (Fig. 2, Table 2), indicating its potential role in the basal defense response of various soybean varieties against HG1.2.3.5.7 infection.
2. Specific KEGG metabolic pathways in the incompatible soybean variety PI437654. Nine specific KEGG metabolic pathways in the incompatible soybean variety PI437654 associated with three differential metabolites including L-arginine, phytosphingosine and D-aspartic acid were identified (Table 2). The specific associated KEGG metabolic pathways included sphingolipid metabolism (ath00600), alanine, aspartate and glutamate metabolism (ath00250), arginine biosynthesis (ath00220), monobactam biosynthesis (ath00261), aminoacyl-tRNA biosynthesis (ath00970), arginine and proline metabolism (ath00330), biosynthesis of secondary metabolites-unclassified (ath00999), ABC transporters (ath02010) and biosynthesis of amino acids (ath01230) (Table 2). Although the tropane, piperidine and pyridine alkaloid biosynthesis KEGG metabolic pathway (ath00960) showed the most obvious difference in the incompatible soybean PI437654 (Fig. 2), it was not specific to this incompatible soybean variety, as it was also present in two compatible soybean varieties, WM82 and ZH13 (Table 2). Among these nine KEGG pathways, sphingolipid metabolism (ath00600), alanine, aspartate and glutamate metabolism (ath00250) and arginine biosynthesis (ath00220) were dramatically changed (Fig. 2; Table 2), suggesting their prevailing roles against HG1.2.3.5.7 infection in the incompatible soybean variety PI437654.

**Table 2:** Analyses of the KEGG metabolic pathways associated with the differential metabolites in the incompatible soybean variety PI437654 and the three compatible soybean varieties, WM82, ZH13 and HF47, infected by HG1.2.3.5.7

Metabolite	KEGG ID	Annotation	PI437654	WM82	ZH13	HF47
16-hydroxyhexadecanoic acid	ath00073	cutin, suberine and wax biosynthesis	1.337*	1.480	1.480	1.402
linolenic acid^a^	ath00592	alpha-linolenic acid metabolism	1.130	1.272	1.272	1.195
	ath01040	biosynthesis of unsaturated fatty acids	0.914	1.052	1.052	0.977
2-oxo-4-methylthiobutanoic acid^a^	ath00270	cysteine and methionine metabolism	/	1.134	1.133	1.058
	ath00966	glucosinolate biosynthesis	/	1.036	1.036	0.961
	ath01210	2-oxocarboxylic acid metabolism	/	0.808	0.808	0.735
isoliquiritigenin	ath00941	flavonoid biosynthesis	/	1.052	1.052	/
isopimaric acid	ath00904^b^	diterpenoid biosynthesis	/	/	/	1.906
ent-kaur-16-en-19-ol						
trans-ferulic acid	ath00940	phenylpropanoid biosynthesis	/	/	/	1.025
phytosphingosine	ath00600	sphingolipid metabolism	1.370	/	/	/
D-aspartic acid	ath00250	alanine, aspartate and glutamate metabolism	1.321	/	/	/
L-arginine ^a^	ath00220	arginine biosynthesis	1.405	/	/	/
	ath00261	monobactam biosynthesis	1.181	/	/	/
	ath00970	aminoacyl-tRNA biosynthesis	1.060	/	/	/
	ath00330	arginine and proline metabolism	0.893	/	/	/
	ath00999	biosynthesis of secondary metabolites - unclassified	0.792	/	/	/
	ath02010	ABC transporters	0.700	/	/	/
	ath01230	biosynthesis of amino acids	0.693	/	/	/
nicotine	ath00960 ^b^	tropane, piperidine and pyridine alkaloid biosynthesis	2.253	/	/	/
pipecolinic acid ^a^				1.088	1.088	/
pipecolinic acid ^a^	ath00310	lysine degradation	1.037	1.177	1.177	/

Note:‘*’ represents the -log_10_ (*p*-value) value, while ‘*p*-value’ represents the *p*-value of each KEGG metabolic pathway. The smaller the *p*-value is, the greater the -log (*p*-value). ‘^a^’ indicates that the same differential metabolite is involved in at least two different KEGG metabolic pathways. ‘^b^’ indicates that two different differential metabolites are involved in the same KEGG metabolic pathway

### Associated genes related to the up-regulated metabolites of the incompatible soybean variety PI437654

To predict the genes associated with the up-regulated differential metabolites of the incompatible soybean variety PI437654, the transcriptome of the incompatible soybean variety PI437654 and the three compatible soybean varieties, WM82, ZH13 and HF47, as well as their corresponding controls, were sequenced, and these transcriptome data were combined with the metabolomic data. The comparative transcriptome analyses identified 15,835 DEGs (6922_UP vs 8913_DOWN), 12,225 DEGs (6001_UP vs 6224_DOWN), 18,362 DEGs (9589_UP vs 8773_DOWN) and 19,528 DEGs (8944_UP vs 10,584_DOWN) in the incompatible soybean variety PI437654 and the three compatible soybean varieties, WM82, ZH13 and HF47, infected by HG1.2.3.5.7, respectively (Fig. S4). As mentioned above, three key metabolites (N-acetyltranexamic acid, nicotine and D,L-tryptophan) that were significantly up-regulated in the incompatible soybean variety PI437654 infected by HG1.2.3.5.7, were classified into two types. One type was specific to the incompatible soybean variety PI437654, and the other type was overlapped but simultaneously dramatically down-regulated in the three compatible soybean varieties.

**Fig. 3:**
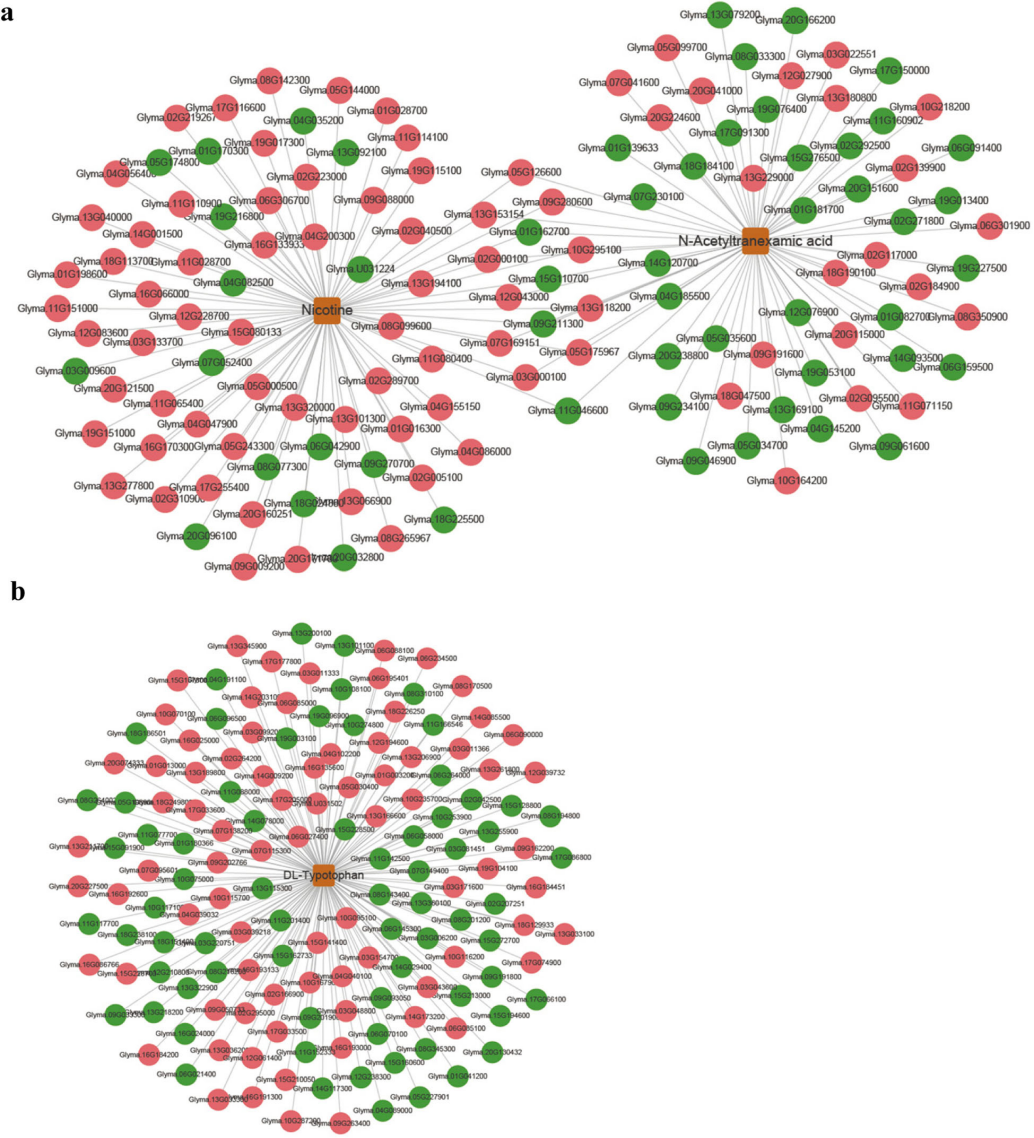
Associated genes related to the significantly up-regulated metabolites in the incompatible soybean variety PI437654 and their associated networks. **a** Genes associated with the significantly up-regulated metabolites specifically present in the incompatible soybean variety PI437654. **b** Genes associated with the significantly up-regulated metabolite D,L-tryptophan, which was significantly up-regulated in the incompatible soybean variety PI437654 but simultaneously dramatically down-regulated in the three compatible soybean varieties, WM82, ZH13 and HF47. Orange squares represent the significantly up-regulated metabolites. Red circles represent positive correlations, while green circles represent negative correlations

**Fig. 4:**
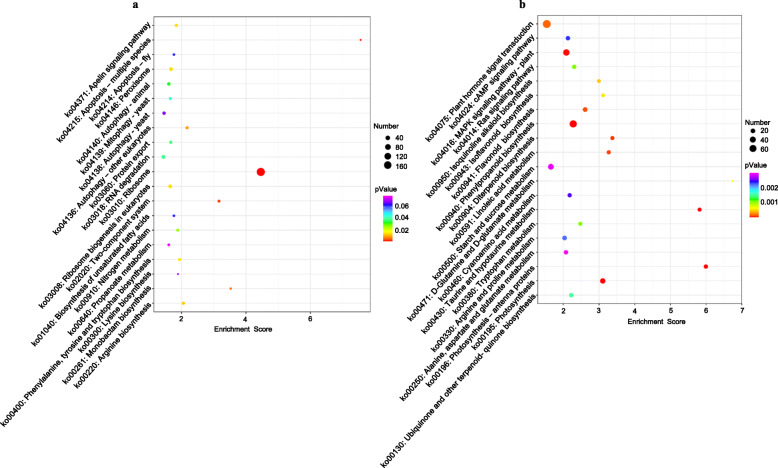
KEGG analyses of the genes associated with the significantly up-regulated metabolites. **a** KEGG analyses of the genes associated with the significantly up-regulated metabolites specifically present in the incompatible soybean variety PI437654. **b** KEGG analyses of the genes associated with the significantly up-regulated metabolite D,L-tryptophan, which was significantly up-regulated in the incompatible soybean variety PI437654 but simultaneously dramatically down-regulated in the three compatible soybean varieties

One of these types was the significantly up-regulated metabolites specifically present in the incompatible soybean variety PI437654, which included N-acetyltranexamic acid and nicotine. The results of combined analyses showed that 14 potentially associated genes (10 positive and four negative correlations) were simultaneously linked to N-acetyltranexamic acid and nicotin, while 68 (52 positive and 16 negative correlations) and 54 (21 positive and 33 negative correlations) associated genes were specifically linked to either nicotin or N-acetyltranexamic acid, respectively (Fig. 3a, Table S3). These associated genes were subjected to GO analyses. The most abundant GO terms in the biological processes category were “translation”, followed by “cytoplasmic translation” and “rRNA modification” (Fig. S5, Table S4). Regarding the cellular component category, the most abundant GO terms were “ribosome”, “cytosolic ribosome”, “cytosolic small ribosomal subunit”, cytosolic large ribosomal subunit” and “nucleolus” (Fig. S5, Table S4). Concerning the molecular function category, the most abundant GO terms were “structural constituent of ribosome”, “mRNA binding”, “rRNA binding”, “RNA binding” and “inorganic cation transmembrane transporter activity” (Fig. S5, Table S4). Accordingly, the associated genes were enriched in the KEGG pathways involving the largest numbers of unigenes, which were “ribosome”, followed by “RNA degradation”, “ribosome biogenesis in eukaryotes”, “peroxisome” and “autophagy” (Fig. 4a, Table S5).

The other type was the significantly up-regulated metabolite in the incompatible soybean variety PI437654 that were simultaneously dramatically down-regulated in the three compatible soybean varieties, which included D,L-tryptophan. Among the top 150 most associated genes, 80 genes exhibited positive correlations, while 70 genes exhibited negative correlations (Fig. 3b, Table S6). Among these associated genes, the most abundant GO terms in the biological processes category were “response to chitin”, followed by the “ethylene-activated signaling pathway” and “defense response” (Fig. S5, Table S7). Regarding the cellular component category, the most abundant GO terms were “extracellular region”, “apoplast” and “photosystem I” (Fig. S5, Table S7). Concerning the molecular function category, the most abundant GO terms were “transcription factor activity, sequence-specific DNA binding”, “heme binding” and “hydroquinone:oxygen oxidoreductase activity” (Fig. S5, Table S7). Accordingly, the associated genes were enriched in the KEGG pathways involving the largest numbers of unigenes, which were “plant hormone signal transduction”, followed by “phenylpropanoid biosynthesis”, “MAPK signaling pathway-plant”, “starch and sucrose metabolism” and “photosynthesis” (Fig. 4b, Table S8).

These results of the combined metabolomic and transcriptomic analyses revealed potential genes associated with the most significantly up-regulated metabolites of the incompatible soybean variety PI437654 and suggested the likely corresponding biological processes, cellular components, molecular functions and KEGG pathways.

## Discussion

In this study, the incompatible soybean variety PI437654 and the three compatible soybean varieties, WM82, ZH13 and HF47, were used to explore the changes in metabolites in soybean roots in response to HG1.2.3.5.7 via LC/MS analyses. In total, 37 differential metabolites and 20 KEGG metabolic pathways were identified, which were divided into three categories: metabolites/metabolic pathways common among all the tested incompatible and compatible soybean varieties; metabolites/metabolic pathways specific to the compatible soybean varieties; metabolites/metabolic pathways specific to the incompatible soybean varieties (Tables 1 and 2). It was found that certain differential metabolites, such as linolenic acid and L-arginine, were probably involved in more than one KEGG metabolic pathway, while other differential metabolites, such as nicotine and pipecolinic acid, were likely involved in the overlapping KEGG metabolic pathways (tropane, piperidine and pyridine alkaloid biosynthesis pathways) (Table 2). However, only 12 differential metabolites (32%) were found to be involved in the predicted KEGG metabolic pathways.

In this study, some basal defense-related potential metabolites, such as linolenic acid and 16-hydroxyhexadecanoic acid, were found in the incompatible soybean variety PI437654 and the three compatible soybean varieties (WM82, ZH13 and HF47) infected by HG1.2.3.5.7, and our metabolomic analysis results were consistent with some previous reports [39–41]. Linolenic acid is involved in alpha-linolenic acid metabolism and the biosynthesis of unsaturated fatty acids [39–43]. Linolenic acid has been found to be associated with abiotic stress responses, such as cold tolerance in wheat [39–41]. Unsaturated fatty acids are considered as a new generation of plant resistance inducers [42, 43]. Amruthesh et al. found that the application of unsaturated fatty acids induced strong resistance to downy mildew in pearl millet [42]. Clay et al. found that the higher contents of unsaturated fatty acids in the nuclear membrane of the resistant species in *Gossypium barbadense* was related to host resistance [43].

The metabolites and associated KEGG metabolic pathways specific to the incompatible soybean variety PI437654 infected with HG1.2.3.5.7 probably play pivotal roles in plant stress, tolerance or resistance based on the related literature [44–61]. L-arginine is the precursor of nitric oxide (NO), and NO is synthesized from L-arginine by NO synthase in the L-arginine-NO pathway. NO is widely considered as an important regulator of cell function and communication in physiological and pathophysiological states [44–46]. NO is a second messenger in the stress response and is involved in plant responses to biotic and abiotic stresses [47, 48]. L-arginine is linked to and involved in many KEGG metabolic pathways, such as arginine and proline metabolism, aminoacyl-tRNA biosynthesis and ABC transporter pathways. Previous studies have indicated that there is a positive correlation between proline accumulation and plant tolerance [49]. Aminoacyl-tRNA synthetases catalyze the connection between amino acids and their homologous tRNAs and constitute a specific set of enzymes ensuring the fidelity of the transfer of genetic information from DNA to proteins [50, 51]. ABC transporters transport material cross membranes by using the energy from ATP hydrolysis [52]. Recently, ABC transporters have attracted increasing attention due to their role in pesticide resistance [53]. Phytosphingosine (PHS) is a member of the sphingosine family that shows fungicidal activity [54]. Castro et al. found that PHS reduced the viability of cells and induced apoptosis in *Neurospora crassa* [55]. D-Aspartic acid participates in alanine, aspartate and glutamate metabolism, while glutamate dehydrogenase (GDH) is a central enzyme in glutamate metabolism [56]. The enhancement of drought resistance in wheat is related to ammonia assimilation and increased NADH-GDH activity under low or high osmotic stress [61]. Nicotine is widely used to kill many kinds of harmful insects [58]. Vänninen at el discovered that nicotine had a strong killing effect on the adults of Miridae species [60]. Nicotine is involved in the biosynthesis of tropane, piperidine and pyridine alkaloids via metabolic pathways [62] and alkaloids have been found to exert obvious inhibitory effects on the sunflower downy mildew fungus *Plasmopara halstedii* [59]. Thus, L-arginine, phytosphingosine, D-aspartic acid and nicotine may be involved in the incompatible response of soybean against soybean cyst nematodes.

In contrast, the differential metabolite 2-oxo-4-methylthiobutanoic acid, which showed overlap among all three compatible soybean varieties but was absent in incompatible soybean variety, was predicted to participate in three KEGG metabolic pathways, including cysteine and methionine metabolism, glucosinolate biosynthesis and 2-oxocarboxylic acid metabolism. Moreover, 2-oxo-4-methylthiobutanoic acid was simultaneously down-regulated in the three compatible soybean varieties (ZH13, WM82 and HF47) infected with HG1.2.3.5.7, which likely resulted in decreased biosynthesis of glucosinolate. It has been reported that glucosinolate is a plant secondary metabolite that exerts a preventive effect against plant pathogens and soil-borne plant pests [63, 64]. Additionally, the KEGG metabolic pathways of flavonoid biosynthesis and diterpenoid biosynthesis, which are reported to be involved in plant resistance to pathogens, were found to be enriched in one or two compatible soybean varieties [65, 66]. These types of differential metabolites and their related KEGG metabolic pathways might play roles in the manipulation of host innate immunity to establish and maintain feeding sites for the development of soybean cyst nematodes [67, 68]. In this study, a local metabolite-calibrated database was used to identify potential differential metabolites, which ensured the accuracy of the results [16, 69, 70]. However, this database also presents potential disadvantages because of lacking many important compounds due to its limited capacity. In this study, the different metabolomic responses of incompatible and compatible soybean varieties to soybean cyst nematode infection were thoroughly characterized, and key differential metabolites and related KEGG metabolic pathways were identified. The likely functions of differential metabolites and related KEGG metabolic pathways were predicted.

In this study, three key metabolites (N-acetyltranexamic acid, nicotine and D,L-tryptophan) that were significantly up-regulated in the incompatible soybean variety PI437654 infected by HG1.2.3.5.7, were identified and classified into two types. The combination of metabolomic analyses with transcriptomic expression profiling aided in predicting potential genes associated with the most significantly up-regulated metabolites of the incompatible soybean variety PI437654 and suggested the likely corresponding biological processes, cellular components, molecular functions and pathways.

## Conclusions

In this study, the main metabolomic differences between the incompatible soybean variety PI437654 and three compatible soybean varieties infected with HG1.2.3.5.7 were characterized. In total, 37 differential metabolites and 20 KEGG metabolic pathways were identified, which were divided into three categories: metabolites/metabolic pathways overlapped among all the tested incompatible and compatible soybean varieties; metabolites/metabolic pathways specific to compatible soybean varieties; metabolites/metabolic pathways specific to incompatible soybean variety. Twelve differential metabolites were found to be involved in the predicted KEGG metabolic pathways. Moreover, 14 differential metabolites such as significantly up-regulated nicotine and down-regulated D-aspartic acid, and their associated KEGG pathways such as tropane, piperidine and pyridine alkaloid biosynthesis, alanine, aspartate and glutamate metabolism, sphingolipid metabolism and arginine biosynthesis were significantly altered and abundantly enriched specifically in the incompatible soybean variety PI437654 and likely played pivotal roles in defending against HG1.2.3.5.7. Moreover, the combination of metabolomic analyses with transcriptomic expression profiling aided in predicting potential associated genes related to the most significantly up-regulated metabolites of the incompatible soybean variety PI437654, and suggested the likely biological processes, cellular components, molecular functions and pathways. To the best of our knowledge, our study was the first to investigate the different responses of incompatible and compatible soybean varieties infected with soybean cyst nematodes by combining metabolomic analyses and transcriptomics. Our results not only identified potential novel metabolites and associated genes involved in the incompatible response of PI437654 to the soybean cyst nematode HG1.2.3.5.7, but also provided new insights into the interactions between soybeans and soybean cyst nematodes.

## Methods

### Soybean seedling preparation and inoculation of soybean cyst nematodes

The seeds of the incompatible soybean variety PI437654 and the three compatible soybean varieties, WM82, ZH13 and HF47, were obtained from China National Gene Bank (http://www.nationalgenebank.org/). Soybean seeds were sterilized by soaking in a 1.0% (w/v) NaClO solution for 5 min [71], and the seeds were then washed with sterilized water to remove residual NaClO. After surface sterilization, the seeds were placed between moist sterilized filter papers at 26 °C. After approximately 48 h, the germinated seeds were transplanted into a soil mixture consisting of 75% (v/v) soil and 25% (v/v) sand and cultured in a greenhouse, where the day/night temperatures were set to 28 °C/26 °C with a photoperiod of 12 h [72–74].

Single cysts of HG1.2.3.5.7 were cultured on the roots of the compatible soybean variety ZH13 grown in an autoclaved soil mixture in a greenhouse at 28 °C/26 °C with a photoperiod of 12 h [13, 73]. The cysts were harvested and placed on 500-mesh (25 μm) sieves in 3 mM ZnCl_2_ solution and then incubated in the dark at 25 °C [38, 73, 75]. The hatched J2s were collected with a glass tube [76]. Fourteen-day-old soybean seedlings were inoculated with 1000 J2s/seedling. At 8 days post inoculation (dpi), the root samples of the incompatible soybean variety PI437654 and the three compatible soybean varieties, WM82, ZH13 and HF47, as well as their corresponding controls were harvested. Each type of root sample was divided into two parts for the following metabolomic and transcriptomic analyses.

### Juveniles staining and cysts counting

Root samples of the incompatible soybean variety PI437654 and the three compatible soybean varieties, WM82, ZH13 and HF47, were collected at 8 dpi, rinsed in water, and then soaked in a 5.25% NaClO solution for 5 min. The soybean samples were then rinsed twice in water for 10 min and 15 min, respectively. The soybean samples were dropped into a boiling 0.01% acid fuchsin solution for 30 s and then rinsed in water after cooling. The samples were transferred to and stored in glycerol [77–80]. The morphology of the HG1.2.3.5.7 nematodes was observed, and their numbers within different soybean roots were counted with a microscope (OLYMPUS-SZ2-ILST).

At 60 dpi, samples of the soil mixture in which the soybean plants were grown were suspended in water, and the soybean roots were processed by washing and rubbing to dislodge the females and cysts. The cysts were extracted by sieving the suspension through nested 20-mesh (850 μm, on the top) over 60-mesh (250 μm, on the bottom) sieves [81, 82]. The numbers of cysts, which were recorded in the form of cysts per plant [81, 82], were counted with a stereoscopic microscope (OLYMPUS-BX53F).

### Untargeted metabolomics using LC/MS

At 8 dpi, the root samples of the incompatible soybean variety PI437654 and the three compatible soybean varieties, WM82, ZH13 and HF47, as well as their corresponding controls were harvested and named PI437654_SCN, PI437654_0, WM82_SCN, WM82_0, ZH13_SCN, ZH13_0, HF47_SCN and HF47_0, respectively. Each soybean root sample was ground into a powder in liquid nitrogen. An 80 mg sample of root powder was then dropped into a 1.5 mL centrifuge tube. Then, 20 μL of an internal standard (0.3 mg/mL L-2- chlorophenylalanine) and 1 mL of 70% methanol (methanol:water =7:3 (volume ratio)) were added to the tube. The tube was placed at − 20 °C for precooling, and the mixture was ground with a grinding machine for 2 min at 60 Hz. Then, ultrasonic extraction was performed at 4 °C for 30 min. After standing at − 20 °C for 20 min, the sample was centrifuged for 15 min at 13,000 rpm at 4 °C, and 200 μL of the supernatant was harvested with a syringe. This procedure was repeated for each sample three times, and the supernatants from the same source were combined. After filtering through a 0.22 μm organic-phase pinhole filter, the corresponding supernatant was subjected to LC-MS analysis. LC-MS analysis was performed on an Agilent 1290 Series UHPLC system coupled to an Agilent 6540 TOF/MS instrument with a Dual AJS ESI source. Each treatment had six repetitions [16, 83].

### Metabolomic analyses

The metabolites were identified by using a local metabolite-calibrated database that contained about 500 metabolites based on the standards of peak information involving retention time and high-precision mass values [16, 69, 70]. Multivariate statistical analyses, including principal component analysis (PCA) and partial least squares discriminant analysis (PLS-DA), were performed using SIMCA-P^+^ 14.0 version software [84] to distinguish the differences in metabolic profiles between the incompatible soybean variety PI437654 and the three compatible soybean varieties, WM82, ZH13 and HF47, infected by HG1.2.3.5.7. The Hotelling’s T2 region, shown as an ellipse in the score plots of the models, defined the 95% confidence interval of the modeled variation. According to the T-test, a variable with a *p*-value < 0.05 was considered as a significantly different variable [83]. Metabolites with VIP (variable importance in the projection) values > 1.0 and *p*-values < 0.05 were selected as differential metabolites [83]. KEGG metabolic pathways were predicted from the differential metabolites showing a *p*-value < 0.05 [83].

### Transcriptomic analyses

Root samples of the same six plants were sampled and randomly divided into two groups for each soybean variety, representing two biological repeats. The total RNA of each sample was extracted using a Plant RNA Kit (TIANGEN, China). Total RNA was quantified and analyzed for quality with an Agilent 2100 Bioanalyzer (Agilent, USA) [85]. One microgram of total RNA with a RNA integrity number (RIN) value above 8.0 was used for the subsequent preparation of libraries [86].

To construct a library [78, 85, 86], mRNA was enriched and purified with oligo (dT)-rich magnetic beads and then broken into short fragments. First- and second-strand cDNAs were synthesized by using the cleaved mRNA fragments as templates. An AxyPrep Mag PCR Clean-up kit (Axygen, USA) was used to further purify the double-stranded cDNA, and End Prep Enzyme Mix (NEB, USA) was used to add adaptors. An AxyPrep Mag PCR Clean-up kit (Axygen, USA) was used to recover fragments of ~ 450 bp. The PCR products were purified, and then the purified PCR products were validated with an Agilent 2100 Bioanalyzer (Agilent, USA) and quantified via Qubit and real-time PCR analysis (Applied Biosystems, USA). RNA sequencing was carried out using a 2 × 150 paired-end (PE) configuration with an Illumina Novaseq 6000 system.

Clean data were obtained by removing the reads that contained adapters and ploy-N sequences and low-quality reads from the raw data [85, 86]. Reference genome and gene model annotation files were downloaded from the SoyBase website (https://soybase.org/GlycineBlastPages/blast_descriptions.php). The paired-end clean reads were aligned to the reference genome by using HISAT [35]. HTSeq was used to calculate the numbers of reads mapped to each gene. The expression of genes was measured in fragments per kb per million fragments (FPKM) values. Genes showing an absolute log_2_ FC (fold change) >0.5 and a pvalue< 0.05 were defined as DEGs, and a software DESeq2 was used to calculate significance [87]. GO (Gene Ontology) analyses were performed by using Blast2GO [86, 88]. KOBAS 2.0 was used to test the statistical enrichment of DEGs in KEGG pathways [35].

### Correlation analyses between metabolome and transcriptome data

Correlation analyses were carried out as previously described [85, 89]. Briefly, Pearson correlation coefficients were calculated for the integration of the metabolome and transcriptome data. The mean contents of metabolites in the incompatible soybean variety PI437654 and the three compatible soybean varieties, WM82, ZH13 and HF47, and the mean transcript abundance of the DEGs together were calculated. One-way analysis of variance (ANOVA) associated with T-test was performed to determine significant differences between groups according to a *p*-value < 0.05. PCA of the total transcriptomic dataset was performed in R (R Core Team) [35]. The coefficients were calculated from the log_2_ FC value of each metabolite and the log_2_ FC value of each transcript. Correlations were determined according to an R^2^ coefficient > 0.9. The relationships between the metabolome and transcriptome data were illustrated by using Cytoscape. The top 150 associated genes were the focuses of subsequent analyses based on the numbers of genes and the complexity of the network involved.

## Supplementary Information

**Additional file 1: Fig. S1.** Development of HG1.2.3.5.7 juveniles in the roots of the incompatible soybean variety PI437654 and the three compatible soybean varieties, WM82, ZH13 and HF47. The root samples of the incompatible soybean variety PI437654 and the three compatible soybean varieties, WM82, ZH13 and HF47, infected by HG1.2.3.5.7 were collected at 8 dpi and then stained with 0.0.1% acid fuchsin solution. The juveniles of HG1.2.3.5.7 within soybean roots were examined by a microscopy. The bar represents 200 μm.

**Additional file 2: Fig. S2.** PCA and PLS-DA of the incompatible soybean variety PI437654 and the three compatible soybean varieties, WM82, ZH13 and HF47, infected with HG1.2.3.5.7 at 8 dpi compared with the results for controls innoculated with water. This chart was generated from the same data represented in Fig. 1, but they were displayed in a different form to show clear separation trends.

**Additional file 3: Fig. S3.** Venn diagrams showing the commonality and uniqueness of the differential metabolites between the incompatible soybean variety PI437654 and the three compatible soybean varieties, WM82, ZH13 and HF47, infected with HG1.2.3.5.7 at 8 dpi compared with the results for controls innoculated with water. The values represent the amounts of specific or overlapping different metabolites.

**Additional file 4: Fig. S4.** Transcriptomic profiles of the number of differentially expressed genes (DEGs) between the incompatible soybean variety PI437654 and the three compatible soybean varieties, WM82, ZH13 and HF47, infected with HG1.2.3.5.7 at 8 dpi compared with the results for controls innoculated with water, respectively. Red represents the number of up-regulated DEGs, while blue represents the number of the down-regulated DEGs.

**Additional file 5: Fig. S5.** GO analyses of the genes associated with the significantly up-regulated metabolites. **(a)** GO analyses of the genes associated with the significantly up-regulated metabolites specifically present in the incompatible soybean variety PI437654. **(b)** GO analyses of the genes associated with the significantly up-regulated metabolite D,L-tryptophan, which was significantly up-regulated in the incompatible soybean variety PI437654 but simultaneously dramatically down-regulated in the three compatible soybean varieties.

**Additional file 6: Table S1**. Numbers of SCN juveniles and cysts in the incompatible soybean variety PI437654 and the three compatible soybean varieties, WM82, ZH13 and HF47, infected by HG1.2.3.5.7.

**Additional file 7: Table S2**. Cumulative interpretation rate of the model in the incompatible soybean variety PI437654 and the three compatible soybean varieties, WM82, ZH13 and HF47, infected by HG1.2.3.5.7.

**Additional file 8: Table S3**. List of the genes associated with the significantly up-regulated metabolites specifically present in the incompatible soybean variety PI437654.

**Additional file 9: Table S4**. GO analyses of the genes associated with the significantly up-regulated metabolites specifically present in the incompatible soybean variety PI437654.

**Additional file 10: Table S5**. KEGG analyses of the genes associated with the significantly up-regulated metabolites specifically present in the incompatible soybean variety PI437654.

**Additional file 11: Table S6**. List of the genes associated with the significantly up-regulated metabolite D,L-tryptophan, which was significantly up-regulated in the incompatible soybean variety PI437654 but simultaneously dramatically down-regulated in the three compatible soybean varieties.

**Additional file 12: Table S7**. GO analyses of the genes associated with the metabolite D,L-tryptophan, which was significantly up-regulated in the incompatible soybean variety PI437654 but simultaneously dramatically down-regulated in the three compatible soybean varieties.

**Additional file 13: Table S8**. KEGG analyses of the genes associated with the metabolite D,L-tryptophan, which was significantly up-regulated in the incompatible soybean variety PI437654 but simultaneously dramatically down-regulated in the three compatible soybean varieties.

## Data Availability

The soybean varieties used in this study were obtained from China National Gene Bank (http://www.nationalgenebank.org/). The data that support the findings of this study have been deposited in the CNSA (https://db.cngb.org/cnsa/) of CNGBdb (China National GeneBank) with accession number CNP0001271.

## Abbreviations

ABC transporters: ATP binding cassette transporters
ANOVA: Analysis of variance
DEGs: Differential expressed genes
dpi: Days post inoculation
FC: Fold change
FPKM: Fragments per kilobase million
GDH: Glutamate dehydrogenase
GO: Gene Ontology
GWAS: Genome-wide association studies
HF47: Hefeng 47
J2s: Juveniles of second stage
KEGG: Kyoto encyclopedia of genes and genomes
LC/MS: Liquid chromatography-tandem mass spectrometry
MAPK: Mitogen-activated protein kinase
NADH-GDH: Reduced form of nicotinamide-adenine dinucleotid-glutamate dehydrogenase
NO: Nitric oxide
PC: Polycarbonate
PCA: Principal component analysis
PCR: Polymerase chain reaction
PE: Paired-end
PHS: Phytosphingosine
PLS-DA: Partial least squares-discrimination analysis
RIN: RNA integrity number
SCN: Soybean cyst nematode
TOF/MS: Time-of-flight mass spectrometer
UHPLC: Ultra high performance liquid chromatography
VIP: Variable important in projection
vs: Versus
WM82: Williams 82
ZH13: Zhonghuang 13
