# Supplementary figures and images for: Combining targeted metabolite analyses and transcriptomics to reveal the specific chemical composition and associated genes in the incompatible soybean variety PI437654 infected with soybean cyst nematode HG1.2.3.5.7
Source: BMC Plant Biol. 2021 May 14;21:217. doi: 10.1186/s12870-021-02998-4 (PMC8120846; doi:10.1186/s12870-021-02998-4)

## Slide 1
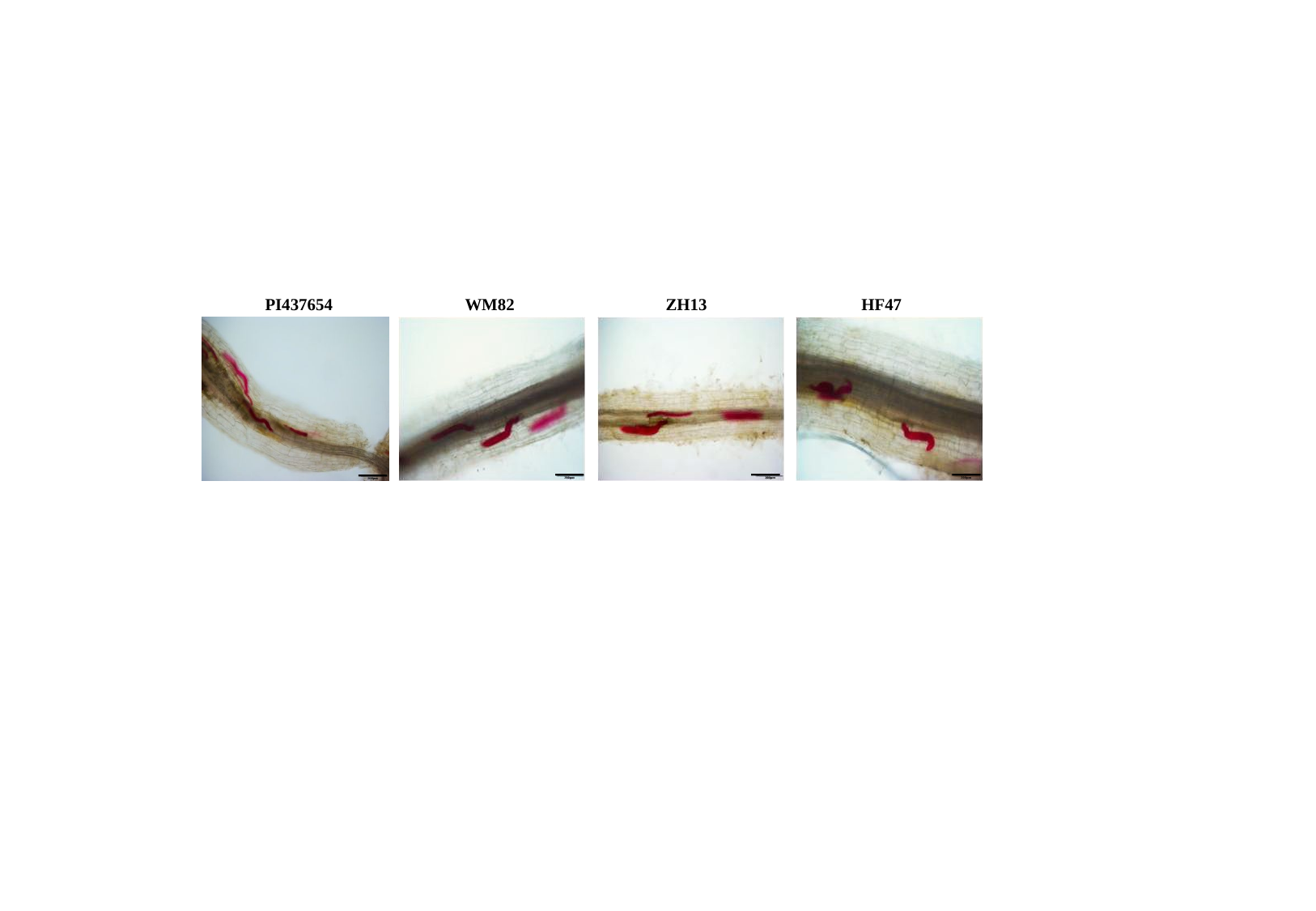

PI437654
WM82
ZH13
HF47
200µm
200µm
200µm
200µm

Supplement: Supplementary file 1 — Additional file 1: Fig. S1. Development of HG1.2.3.5.7 juveniles in the roots of the incompatible soybean variety PI437654 and the three compatible soybean varieties, WM82, ZH13 and HF47. The root samples of the incompatible soybean variety PI437654 and the three compatible soybean varieties, WM82, ZH13 and HF47, infected by HG1.2.3.5.7 were collected at 8 dpi and then stained with 0.0.1% acid fuchsin solution. The juveniles of HG1.2.3.5.7 within soybean roots were examined by a microscopy. The bar represents 200 μm. [file 12870_2021_2998_MOESM1_ESM.pptx]

## Slide 1
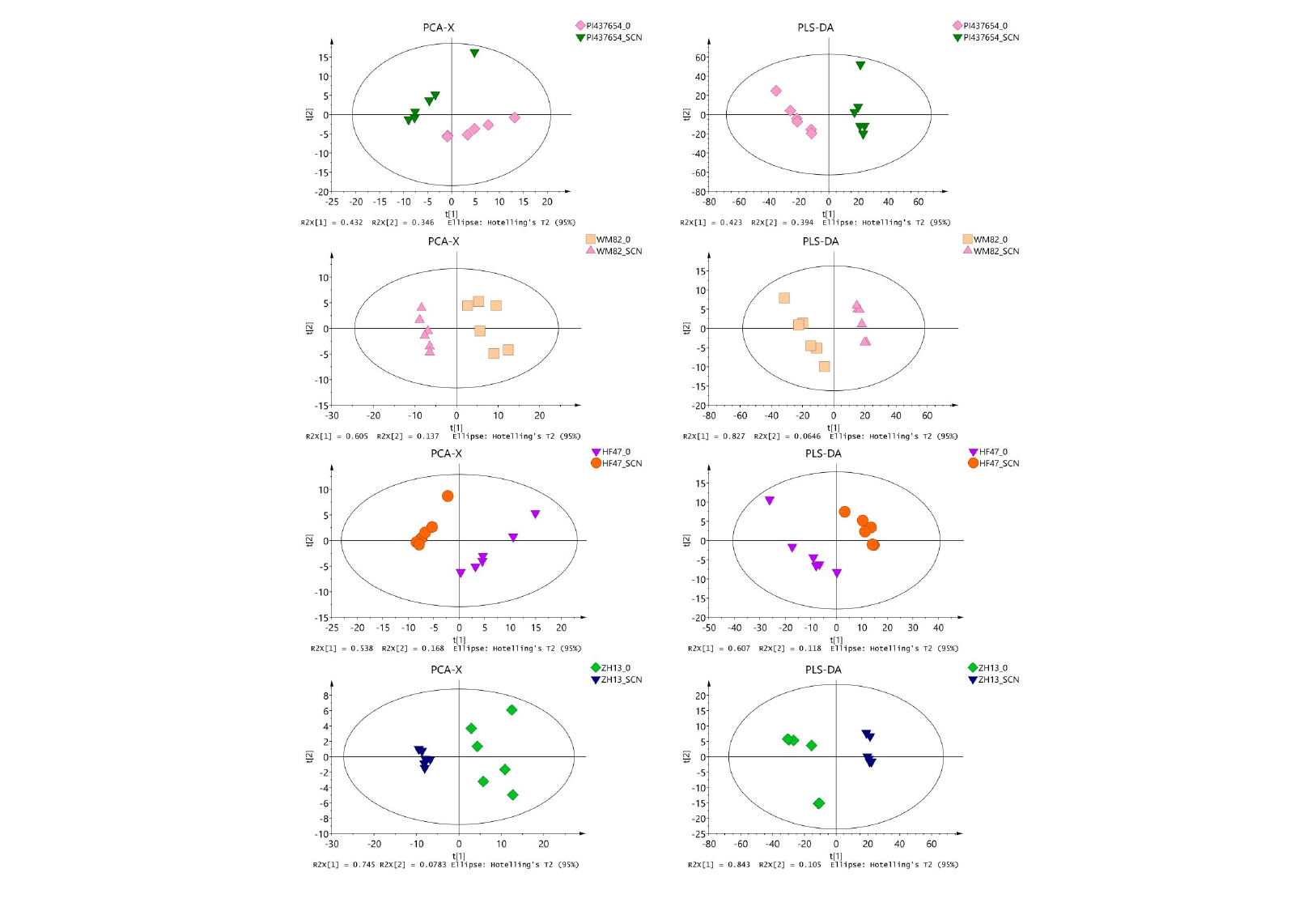

Supplement: Supplementary file 2 — Additional file 2: Fig. S2. PCA and PLS-DA of the incompatible soybean variety PI437654 and the three compatible soybean varieties, WM82, ZH13 and HF47, infected with HG1.2.3.5.7 at 8 dpi compared with the results for controls innoculated with water. This chart was generated from the same data represented in Fig. 1, but they were displayed in a different form to show clear separation trends. [file 12870_2021_2998_MOESM2_ESM.pptx]

## Slide 1
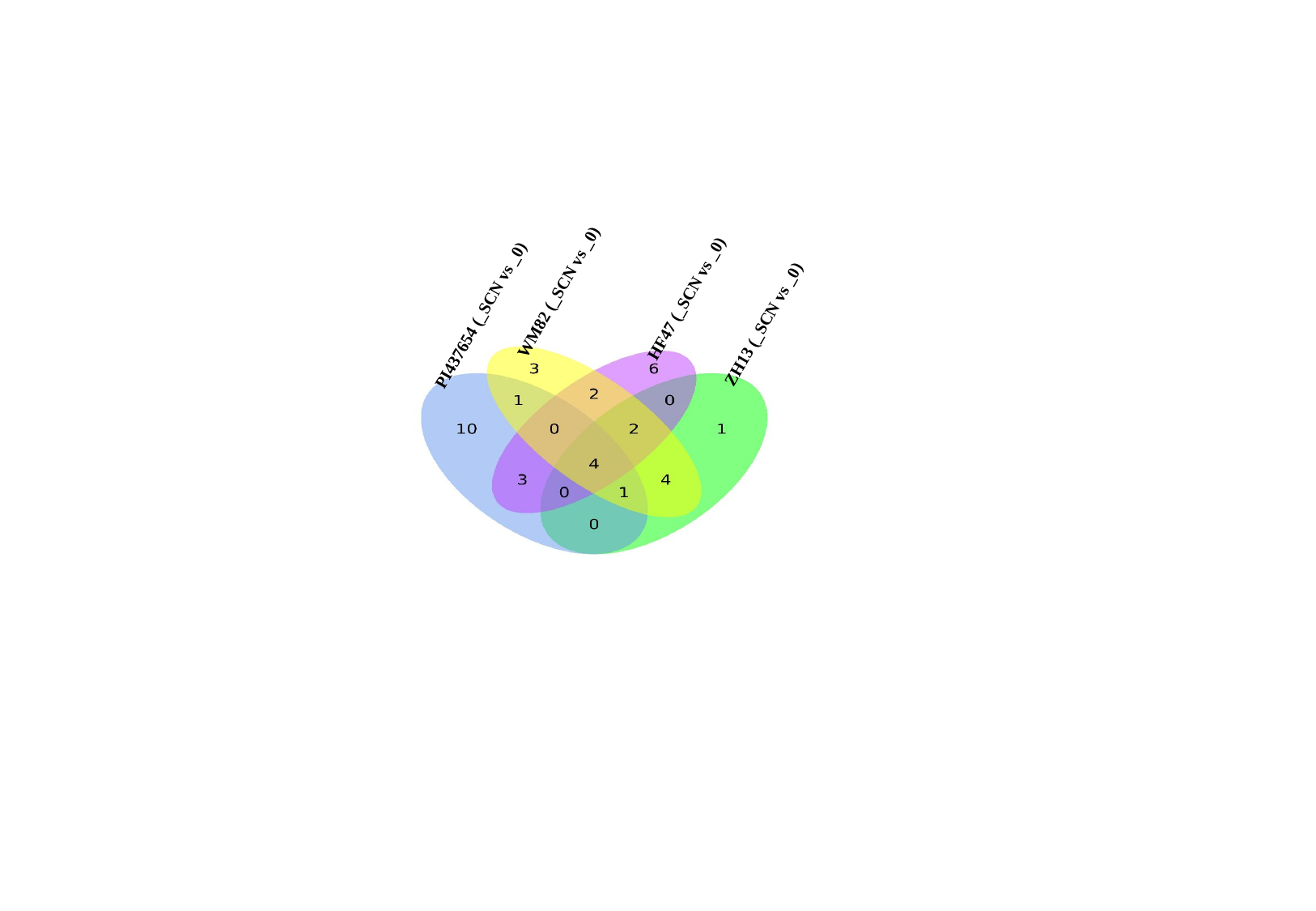

WM82 (_SCN vs _0)
HF47 (_SCN vs _0)
PI437654 (_SCN vs _0)
ZH13 (_SCN vs _0)

Supplement: Supplementary file 3 — Additional file 3: Fig. S3. Venn diagrams showing the commonality and uniqueness of the differential metabolites between the incompatible soybean variety PI437654 and the three compatible soybean varieties, WM82, ZH13 and HF47, infected with HG1.2.3.5.7 at 8 dpi compared with the results for controls innoculated with water. The values represent the amounts of specific or overlapping different metabolites. [file 12870_2021_2998_MOESM3_ESM.pptx]

## Slide 1
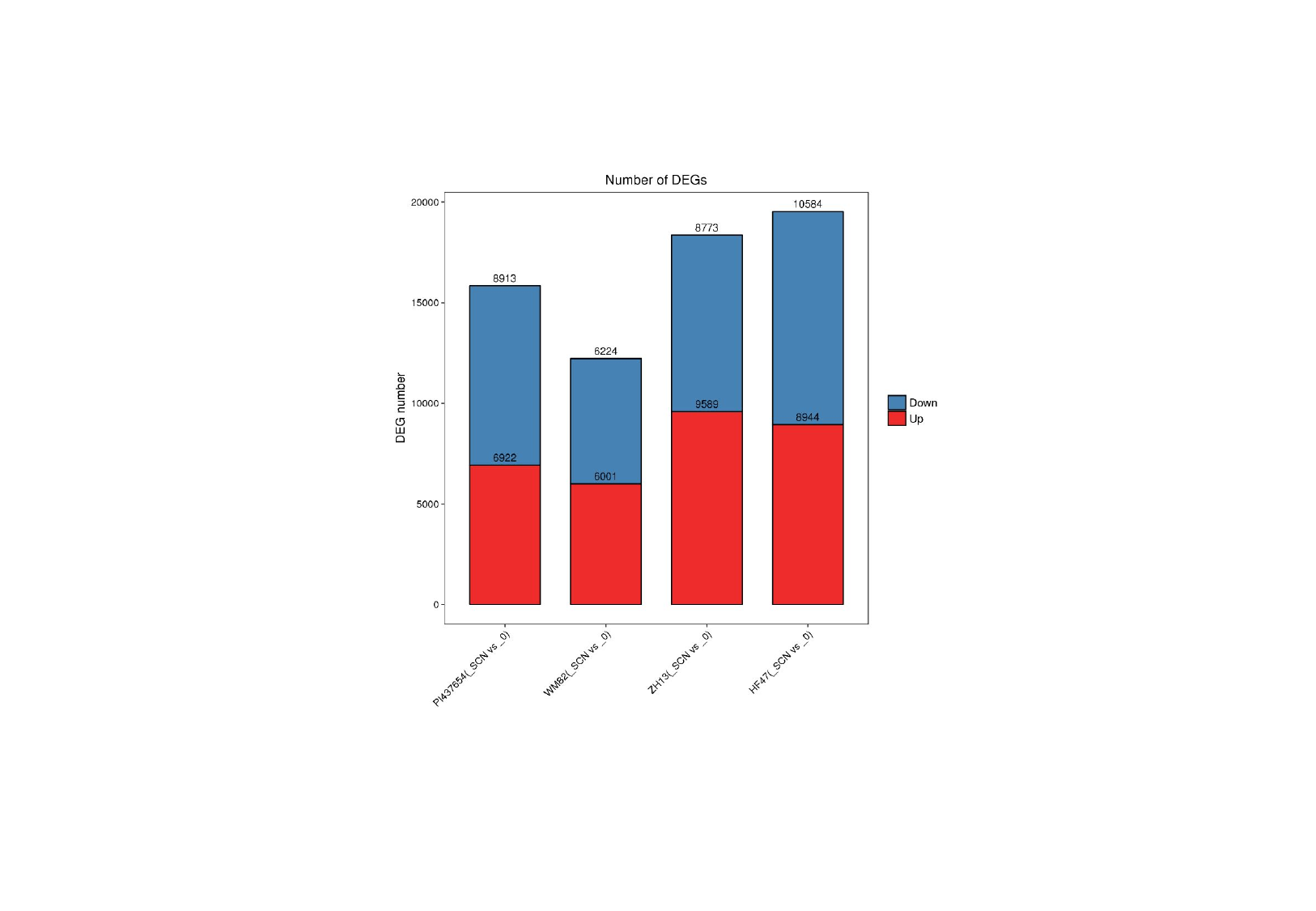

Supplement: Supplementary file 4 — Additional file 4: Fig. S4. Transcriptomic profiles of the number of differentially expressed genes (DEGs) between the incompatible soybean variety PI437654 and the three compatible soybean varieties, WM82, ZH13 and HF47, infected with HG1.2.3.5.7 at 8 dpi compared with the results for controls innoculated with water, respectively. Red represents the number of up-regulated DEGs, while blue represents the number of the down-regulated DEGs. [file 12870_2021_2998_MOESM4_ESM.pptx]

## Slide 1
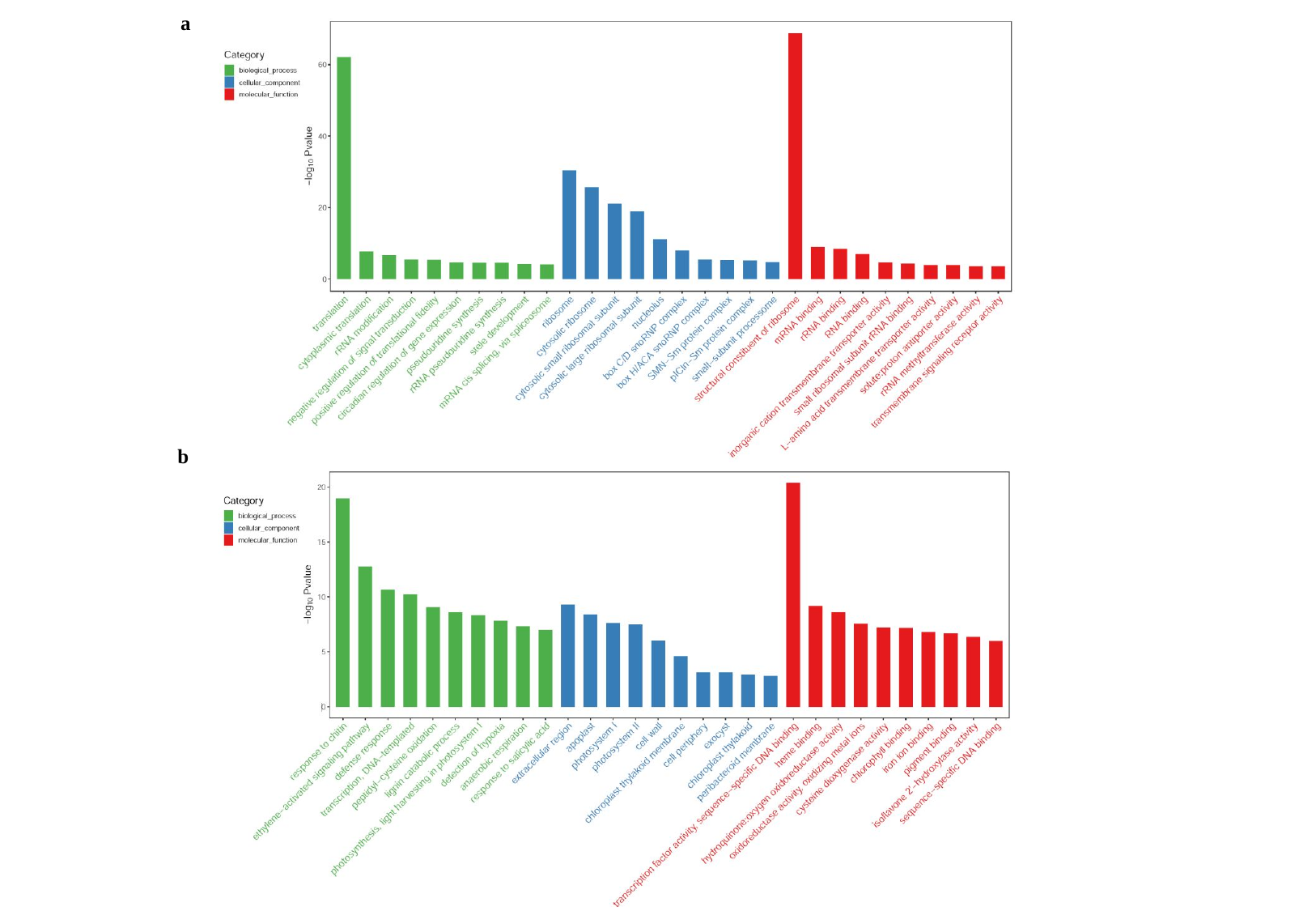

a
b

Supplement: Supplementary file 5 — Additional file 5: Fig. S5. GO analyses of the genes associated with the significantly up-regulated metabolites. (a) GO analyses of the genes associated with the significantly up-regulated metabolites specifically present in the incompatible soybean variety PI437654. (b) GO analyses of the genes associated with the significantly up-regulated metabolite D,L-tryptophan, which was significantly up-regulated in the incompatible soybean variety PI437654 but simultaneously dramatically down-regulated in the three compatible soybean varieties. [file 12870_2021_2998_MOESM5_ESM.pptx]
