# Supplementary material for: Combining targeted metabolite analyses and transcriptomics to reveal the specific chemical composition and associated genes in the incompatible soybean variety PI437654 infected with soybean cyst nematode HG1.2.3.5.7
Source: BMC Plant Biol. 2021 May 14;21:217. doi: 10.1186/s12870-021-02998-4 (PMC8120846; doi:10.1186/s12870-021-02998-4)
Supplement: Supplementary file 6 — Additional file 6: Table S1. Numbers of SCN juveniles and cysts in the incompatible soybean variety PI437654 and the three compatible soybean varieties, WM82, ZH13 and HF47, infected by HG1.2.3.5.7. [file 12870_2021_2998_MOESM6_ESM.docx]

**Table S1.** Numbers of SCN juveniles and cysts in the incompatible soybean variety PI437654 and the three compatible soybean varieties, WM82, ZH13 and HF47, infected by HG1.2.3.5.7

| **Variety** | **No. of J2s** | **No. of J3s** | **No. of cysts** |
| --- | --- | --- | --- |
| PI437654 | 57.5±1.5^b^ | 71±9^a^ | 1±0.6^a^ |
| WM82 | 60±4^b^ | 86±8^a^ | 114±13.5^c^ |
| ZH13 | 31.5±8.5^a^ | 86±10^a^ | 205±4.1^b^ |
| HF47 | 68.5±1.5^b^ | 148±21^b^ | 250±1.2^b^ |

Soybean roots were collected at 8 days post inoculation (dpi) of HG1.2.3.5.7, and the juveniles within soybean roots were stained with 0.01% acid fuchsin solution. Cysts were harvested at 60 dpi, and their numbers were counted with a stereoscopic microscopy. The data were analyzed by using T-test (p-value ≤0.05, n=3). Different letters (a, b and c) above the numbers denoted significant differences.
