# Supplementary material for: Combining targeted metabolite analyses and transcriptomics to reveal the specific chemical composition and associated genes in the incompatible soybean variety PI437654 infected with soybean cyst nematode HG1.2.3.5.7
Source: BMC Plant Biol. 2021 May 14;21:217. doi: 10.1186/s12870-021-02998-4 (PMC8120846; doi:10.1186/s12870-021-02998-4)
Supplement: Supplementary file 7 — Additional file 7: Table S2. Cumulative interpretation rate of the model in the incompatible soybean variety PI437654 and the three compatible soybean varieties, WM82, ZH13 and HF47, infected by HG1.2.3.5.7. [file 12870_2021_2998_MOESM7_ESM.docx]

**Table S2.** Cumulative interpretation rate of the model in the incompatible soybean variety PI437654 and the three compatible soybean varieties, WM82, ZH13 and HF47, infected by HG1.2.3.5.7.

| **Title** | **Type** | **N** | **R^2^X(cum)** | **R^2^Y(cum)** | **Q^2^(cum)** |
| --- | --- | --- | --- | --- | --- |
| PI437654 (_SCN vs _0) | PCA | 12 | 0.776 | / | / |
| WM82 (_SCN vs _0) | PCA | 12 | 0.739 | / | / |
| ZH13 (_SCN vs _0) | PCA | 12 | 0.823 | / | / |
| HF47 (_SCN vs _0) | PCA | 12 | 0.708 | / | / |
| PI437654 (_SCN vs _0) | PLS-DA | 12 | 0.817 | 0.949 | 0.807 |
| WM82 (_SCN vs _0) | PLS-DA | 12 | 0.891 | 0.986 | 0.955 |
| ZH13 (_SCN vs _0) | PLS-DA | 12 | 0.964 | 0.988 | 0.962 |
| HF47 (_SCN vs _0) | PLS-DA | 12 | 0.825 | 0.99 | 0.942 |

Note: N, Numbers of samples analyzed; R^2^X, Interpret-ability of X variable; R^2^Y, Interpret-ability of Y variable; Q^2^, Predictable value of the model. The closer R^2^Y and Q^2^ values were to 1, the better the PLS-DA model interpreted and predicted the differences between the two groups of samples.
